# Understanding older adults' intention to use patient-accessible electronic health records: Based on the affordance lens

**DOI:** 10.3389/fpubh.2022.1075204

**Published:** 2023-01-24

**Authors:** Xindi Wang, Yuxiang Chris Zhao

**Affiliations:** School of Economics and Management, Nanjing University of Science and Technology, Nanjing, China

**Keywords:** older adults, patient-accessible electronic health records, attachment theory, affordance lens, usage intention

## Abstract

**Background:**

Given the aging population and the rapid development of the digital society, concerns about promoting older adults' health skills are increasing. Patient-accessible electronic health records (PAEHRs) are implemented globally for aging health safeguards. The demand for using health-related information communication technologies (ICTs) among older adults and the factors that promote their usage intention of PAEHRs need to be studied.

**Methods:**

Drawing upon affordance theory, we constructed a research model that integrates four affordance types, aggregation, interactivity, collaboration, and communication, to identify the effects of affordances and attachment to platforms and doctors that contribute to older adults' usage intention on PAEHRs. Online survey data from 498 older adults (above 60 years) were collected and analyzed using partial least square-structural equation modeling.

**Results:**

Our findings demonstrated how PAEHR's affordances facilitate older adults' attachment to platforms and doctors. We found that aggregation (γ = 0.417, *P* < 0.001) and interactivity (γ = 0.397, *P* < 0.001) can positively influence older adults' attachment to the PAEHR platform, and collaboration (γ = 0.407, *P* < 0.001) affordance can positively influence older adults' attachment to doctors on the PAEHR platform. Furthermore, seniors' attachment to the platform (γ = 0.598, *P* < 0.001) and attachment to the doctor (γ = 0.156, *P* < 0.01) can both positively influence their usage intention, and attachment to the platform had a positive relationship with attachment to doctors (γ = 0.230, *P* < 0.001) on the PAEHR.

**Conclusion:**

This study enriched the understanding of elders' attachment to doctors on PAEHRs and contributed to the literature on health-related ICT usage targets of older adults. Our findings also shed light on inspiring operators of health-related ICTs to formulate appropriate strategies for aging-friendly design to guide older adults to adopt health-related ICTs in their everyday health information practices.

## Introduction

Health care organizations are faced with supporting clinical decision-making, empowering consumers, and advancing science through information technology. Given the aging population and concerns about promoting the personal health skills of older adults, patient-accessible electronic health records (PAEHRs) are implemented globally to address aging problems, including doctor–patient relationships and health management (1). However, due to the narrow channel of health knowledge popularization and its limited target population, the scope of the audience for health education has not been extended to elder consumers. Human-computer interaction (HCI) research on aging has highlighted the possibilities for technology to promote older adults' health and wellbeing (2). Wiljer et al. (3) showed that most renowned experts at the PAEHR workshop agreed that accessing the EHR is a patient's fundamental right. Nevertheless, there was little agreement on exactly how access should be provided.

While many older adults resist digital technology (4), they are also critical adopters of health-related ICTs. With the aging and digitalization of society, the field of information behavior has gradually begun to focus on the needs of older adults' population to use health-related information systems (5, 6). For example, Faiola (7) verified the important role of mHealth in empowering the elders to participate in health care and decision-making. Hoque (8) identified that performance expectations, effort expectations, and social influence are significant influencing factors that promote the adoption of mHealth by older adults based on the UTAUT model. To promote the adoption of health-related ICTs among older adults, Pan (9) suggested that mHealth should reduce older adults' use costs and help them benefit from social, informational, and emotional gratification. Considering that elders' attachment to PAEHRs is not only pertinent to their relationship with doctors but also relates to platforms' technological features, the concept of “affordances” from ecological psychology can explain their sustained engagement with health-related ICTs. Affordances are the possibilities of goal-directed actions that an object provides to a goal-oriented actor (10). Many researchers have adopted the concept of affordance to investigate the actualization of EHR among health care organizations. For example, Strong et al. (11) recognize affordance theory as a lens through which to study the possibilities afforded to the medical group for accomplishing their goals through an EHR system. Vos et al. (12) examined how the collaborative affordances of an EHR are actualized in its use by health care professionals and pointed out that the optimal actualization of EHRs' collaborative affordances in hospitals requires organizational, technical, and behavioral adaptations. Burton-Jones and Volkoff (13) investigated effective use in the context of community-care EHR systems and how a network of affordances supports the achievement of organizational goals.

Recently, research on PAEHR targeted the viewpoints of patients and health care professionals (14). Some of them focused on health care professionals' perceived effects of PAEHR 6 years after launch (15, 16), but most research concentrated on patients' perceptions of health-related engagement and empowerment (17). Burke Redmond et al. (18) configured a subset of a web-based multimedia patient-accessible electronic health record for patient and family access and suggested that the electronic health record could become a useful tool for health information exchange. Moll and Rexhepi (19) investigated patients in Sweden for the long-term effects of PAEHRs on their communication with health care professionals and their involvement in the care process. Rexhepi et al. (20, 21) provided an in-depth understanding of cancer patients' attitudes and experiences with online medical records and indicated that more patients in the near future might prefer to receive bad news through PAEHR, which can reduce anxiety instead of causing negative emotions. Nurgalieva et al. (22) explored patients' perspectives on sharing their health data through PAEHR and surprisingly found that older patients and patients with lower educational levels share more frequently. Eriksson-Backa et al. (23) studied older adults' experiences of using the PAEHRs' associated portal and other electronic health services and found that improving security, usability and additional information and functions might increase their effective use.

To date, previous studies have provided inadequate insights into PAEHR usage intention among older adults and have also been limited in terms of users' attachment to doctors and platforms relating to PAEHRs. These two gaps point to a critical need for a theory-informed analytical understanding of how elder individuals' perceptions of PAEHRs' function affect their usage intention from the affordance lens. The first objective of our study was to identify the antecedents of older adults' intention to use PAEHRs by considering attachment theory and affordance perspectives, developing a research model to illustrate how the affordances of PAEHRs can facilitate elders' attachment to doctors and platforms and the influencing mechanism of attachment on usage intention. The second objective of our study was to explore the possible existence of relationships between the older adult's perception of PAEHRs' affordance, elders' attachment to the platform due to feature-rich integration and interaction with PAEHRs, and elders' attachment to doctors due to communication and collaboration between them. Based on affordance theory and attachment theory, we embarked on an empirical study of PAEHR usage intention among the older adult population. In particular, this study addressed three research questions:

RQ1—How could PAEHRs' affordance influence older adults' attachment to doctors and platforms?
RQ2—How could older adults' attachment to doctors and platforms influence their usage intention of PAEHRs?
RQ3—How does older adults' attachment to platforms' technological features affect their attachment to doctors on PAEHRs?

## Research model and hypothesis development

### Attachment and usage intention

Organizational scholars have defined attachment as the psychological state of the employee's relationship with their organization. In the field of ICT, it can be defined as an attachment to online group members and the online platform they were using (24). Older adults can form an attachment to a particular object (25). The attachment to a unique ICT such as PAEHRs, or rather the platform that can provide actions has stimulated the enthusiasm of older adults. Especially when people receive positive feedback in the process of interacting with PAEHRs (for example, making medical decisions or learning health-related knowledge), they tend to generate positive emotions such as confidence and trust, which will encourage them to use PAEHRs more actively. Thus, we hypothesized the following:

*H1: Object attachment to the platform of PAEHRs positively influences the usage intention of older adults*.

Elders' relationship with doctors on PAEHRs can also be a factor influencing their intention of health management (26). We thought the effect of older adults' emotional attachment reflects an affective bond between patients and doctors on EHR usage intention. Patients' emotional attachment to doctors drives their usage intention, as they revisit the doctors on the platform (27). Elders' emotional attachment has a mediating role in driving their routinized use of social media (28). In the context of patient-accessible electronic health records, we hypothesized the following:

*H2: Emotional attachment to doctors on PAEHRs positively influences the usage intention of older adults*.

In the context of PAEHRs, we also discuss the effect of older adults' attachment to a platform on their attachment to doctors who provide health care services on it. Currently, it is necessary to rely on ICTs to communicate with doctors for health-related purposes (29). Attachment to the platform drives people to manage their health and enhances patient–doctor emotional bonds on PAEHRs. The patients who used personal health records reported more positive feedback, such as maintaining contact with their doctors (30). The stronger people's dependence on PAEHRs, the more eager they are to engage with their health issues, thereby influencing users' attachment to doctors through communication and collaboration on the platform. Thus, we hypothesized the following:

*H3: Older adults' object attachment to the platform of PAEHRs positively influences their emotional attachment to doctors*.

### Affordances and attachment

The affordances that provide user-object interactions have been informed design decisions for facilitating users' object attachment (25), which implies that EHR affordance should be taken into account as a key factor that influences people's attachment to the platform. Drawing upon affordance theory, we constructed a research model that integrates aggregation affordance, interactivity affordance, collaboration affordance, and communication affordance to test how these affordances affect elders' attachment to the EHR platform. The aggregation affordance and interactivity affordance are proposed from the perspective of the platforms' technological features.

The aggregation affordance of PAEHRs refers to integrating other assistive functions in addition to the electronic medical record. Aggregation affordance provides users with multiple function combinations to complete the task according to their wishes (31), which affords users access to multiple functions within a single application. According to the efficient integration of required functions, which can be operated by users, the PAEHR with aggregation affordances can motivate people to enhance their socialization and meaningful life (32). Thus, we hypothesized the following:

*H4: Aggregation affordance positively influences older adults' attachment to the platform of PAEHRs*.

PAEHRs' interactivity affordance enables users to know what the PAEHR is and how it works (33), including accessing, reviewing (medical advice), and editing (height, weight, or blood pressure) individual electronic health records or blocking certain medical records from access by other medical staff. With greater interactivity enabled through platforms, the PAEHR provides chances for individuals to master their own abilities or those given by affordances. The elders can become increasingly habitually dependent on these apps for recording or reviewing health records. Thus, we hypothesized the following:

*H5: Interactivity affordance positively influences older adults' attachment to the platform of PAEHRs*.

Collaboration and communication affordances are proposed from the perspective of the doctor–patient relationship. The collaboration affordance enables users to work together with doctors through PAEHR's coordination features to achieve their goals (34). Wallberg et al. (35) found sound evidence that the majority of patients preferred a collaborative role in treatment decisions, and some of them preferred a passive form of collaboration in which the doctor made the final treatment decision. Doctor–patient collaboration is positively linked to greater fulfillment of patient expectations (36), which might lead elders to form a strong emotional attachment to doctors who can show a cooperative relationship with them. Thus, we hypothesized the following:

*H6: Collaboration affordance positively influences older adults' attachment to doctors on PAEHRs*.

Integrating communication about health information within health care systems empowers customers with the accessibility of professionals' assistance, which is a vital part of patient treatment (37). The communication affordance allows users to send messages to doctors or contact health care medical staff electronically and ask questions about medical records (33). The great quality of doctor–patient communication increases the trust between doctors and patients (38) and constructs a harmonious physician–patient relationship, which leads to elders' compliance and satisfaction with doctors. Thus, we hypothesized the following:

*H7: Communication affordance positively influences older adults' attachment to doctors on PAEHRs*.

**Figure 1:**
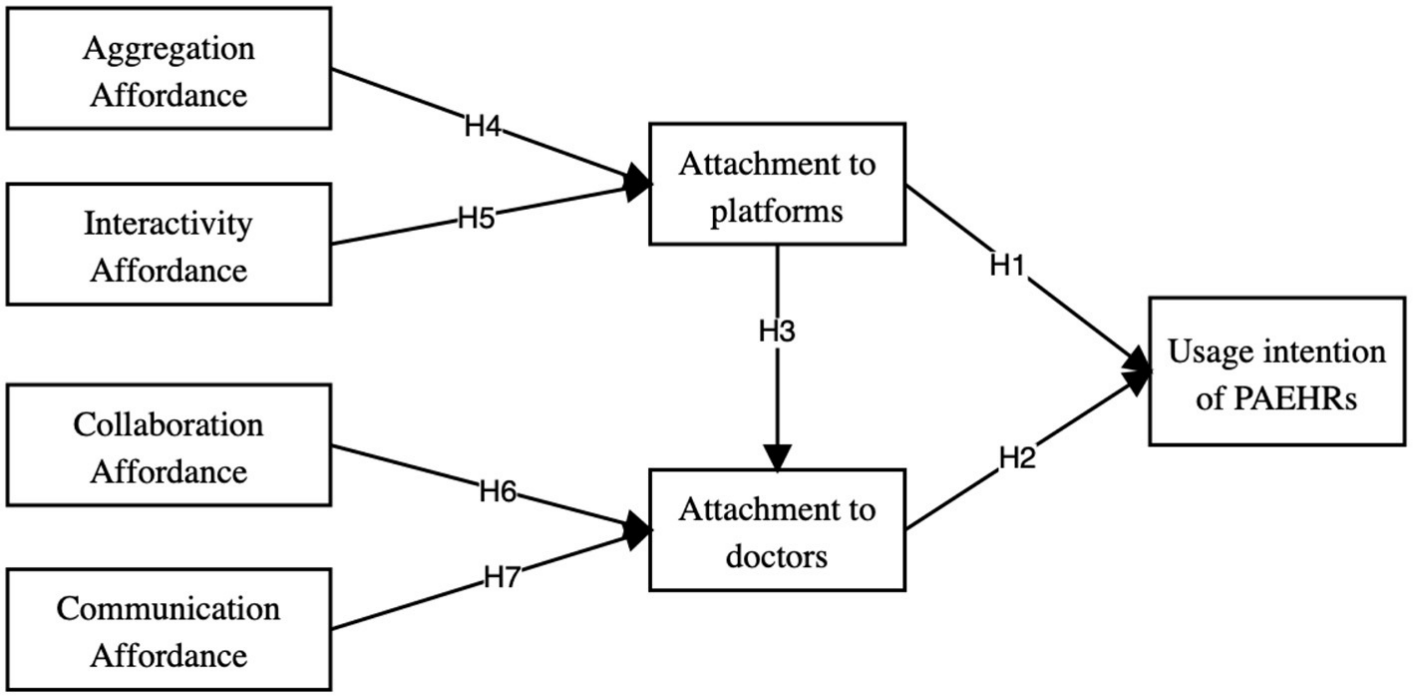
Research model.

The research model is presented in Figure 1 and is based on the attachment theory, affordance, and usage intention literature. We aim to examine the effect of PAEHRs' affordance (i.e., aggregation affordance, interactivity affordance, collaboration affordance, and communication affordance) on older adults' perceived attachment (i.e., attachment to platform and attachment to doctors) and their usage intention. We also posited that older adults' attachment to platform routes contributes to their attachment to doctors on PAEHRs, which further influences usage intention.

## Methods

### Measures

All construct items in this study were adapted from existing studies. Each item was measured using a seven-point Likert scale ranging from 1 (strongly disagree) to 7 (strongly agree). There were two stages of questionnaire formation. Initially, we invited six elder consumers with health-oriented experience in mobile applications from the aging community to participate in the initial questionnaire. We revised the questionnaire and obtained the final version based on their feedback. The construct items and references are detailed in Table 1.

**Table 1:** Research items and references.

**Constructs**	**Items**	**Source**
Aggregation affordance	AA1: I would like to review all my health records (i.e., blood pressure, blood glucose) on the PAEHR. AA2: I expect the PAEHR to integrate multiple functions (i.e., appointment registration, online consultation). AA3: I expect the PAEHR to integrate my physical examination reports, medical advice, and other information from different medical institutions.	(31, 32)
Interactivity affordance	IA1: I expect to fully access several sections of the PAEHR system (i.e., health content, medical records). IA2: I would like to record my basic personal information (i.e., height, weight, exercise history, sleep duration) on the PAEHR. IA3: I expect to choose what I want to browse on the PAEHR.	(33, 39)
Collaboration affordance	COL1: I look forward to treating my doctor as a friend and working with them to solve health problems. COL2: I look forward to treating my doctor as a partner and working with them to make health-related plans (i.e., diet plan, exercise plan). COL3: I hope to make efforts for my treatment and recovery by deep thinking with a doctor.	(36, 40)
Communication affordance	COM1: I look forward to discussing health issues with doctors on PAEHRs. COM2: I look forward to being able to ask doctors about health problems on PAEHRs. COM3: I hope that when I am in doubt during my treatment, I can ask my doctor for advice through PAEHRs.	(33, 38)
Attachment to platform	AP1: I think it is important to record my health information (i.e., blood pressure, blood lipids, sleep quality) on PAEHRs. AP2: I think it is important to keep an electronic health record of my medical history, prescriptions, or physical reports. AP3: I'm interested in using PAEHRs.	(30, 41)
Attachment to doctors	AD1: I want to get along well with doctors on PAEHRs. AD2: I look forward to getting closer to doctors on PAEHRs. AD3: Doctors' words have an impact on my thoughts.	(24, 27)
Usage intention of PAEHRs	UI1: I will use PAEHRs to record personal health information. UI2: I will upload my medical prescriptions and other health contents to the PAEHR. UI3: I will keep consultation notes in PAEHRs' electronic health record.	(30, 31)

### Data collection and sample

To examine the proposed research model, we conducted an online survey focused on older adult users over 60 in China and adopted the snowball sampling approach for data collection. People over 60 years of age were selected as potential respondents in the current study for the following reasons. First, according to previous studies, people over 60 are defined as older adults (29). This age group is also usually considered in studies related to health care services among older adults (42). Second, chronic disease prevalence exists among people over age 60; thus, this population segment could have more potential needs and concerns for personal health management.

Finally, a total of 498 questionnaires were collected. Table 2 summarizes the demographic information of the 498 participants, 48.2% were males and 51.8% were females. The age of the participants mostly ranged from 60 to 65. In total, 92.6% thought they were in good health (chose “Excellent,” “Very well,” and “Good” in the health status option). Two participants reported that their health status was very poor. We set up a multiple-choice question and found that “Recording basic health information (height, weight, blood pressure, blood lipids, etc.)” were selected most frequently as the main contact activity on PAEHRs.

**Table 2:** Demographic information of participants (*N* = 498).

**Measure**	**Items**	**Frequency**	**Percentage (%)**
Gender	Male	240	48.2
	Female	258	51.8
Age	60–65	256	51.4
	66–70	145	29.1
	71–75	68	13.7
	76–80	21	4.2
	Over 81	8	1.6
Health status	Excellent	52	10.4
	Very well	213	42.8
	Good	196	39.4
	Poor	35	7.0
	Very poor	2	0.4
Main activities on PAEHRs (multiple choices)	Recording basic health information (height, weight, blood pressure, blood lipids, etc.)	423	84.9
	Uploading/accessing health data (heart rate, sleep time) from smart devices such as smart bracelets	242	48.6
	Recording exercise duration and caloric expenditure	172	34.5
	Uploading/reviewing medical reports, physical examination reports	328	65.9
	Reviewing previous medical records	286	57.4
	Consulting doctors online (sending messages, instant communication)	189	38.0
	Others	16	3.2

## Results

In this study, structural equation modeling (SEM) was adopted to test the proposed hypotheses. We utilized partial least squares (PLS) to test the proposed model, which is a suitable method for the validation of exploratory studies and relatively complicated models (43). SmartPLS 3.3.3 was employed to test both the measurement and structural models. The statistical significance levels of the structural model path coefficients were verified using the bootstrapping technique.

### The measurement model

The reliability and validity of the research model were evaluated through multiple data analyses. Reliability is a test of the consistency or stability of survey data. The value of Cronbach's alpha (α) and the composite reliability (CR) were used to examine the internal consistency of the conceptual model constructs. Cronbach's alpha values exceeded the recommended level of 0.7, indicating acceptable reliability. The CR values exceeded the recommended level of 0.8, indicating good reliability (44).

Furthermore, the average variance extracted (AVE) was used to measure the convergent and discriminant validity of the research model. First, the AVE values of all constructs exceed the 0.5 thresholds, suggesting qualified convergence validity and demonstrating that items can effectively show the corresponding constructs (45). The reliability and convergence validity results are detailed in Table 3. Moreover, the diagonal of Table 4 is the square root of AVE, and its values were all higher than the correlation of inner constructs, confirming that the measurement model has good discriminant validity. Our results also showed (see Table 5) that item loadings on their construct were higher than the cross-loadings on any other construct, and all item loadings were larger than the required value of 0.7. These results thus also suggest that convergent validity and discriminant validity are satisfied (46). Finally, we conducted Harman's single-factor test on the ten constructs in the research model. The results showed that the most covariance explained by one factor is 39.236%, indicating that common method biases are unlikely threats to our results (47).

**Table 3:** Reliability and convergent validity.

**Construct**	**Composite reliability**	**AVE**	**Cronbach's alpha**
AA	0.839	0.636	0.717
IA	0.838	0.633	0.710
COL	0.845	0.646	0.726
COM	0.863	0.677	0.763
AP	0.869	0.688	0.773
AD	0.843	0.642	0.722
UI	0.851	0.656	0.737

**Table 4:** Discriminant validity.

**Construct**	**AA**	**IA**	**COL**	**COM**	**AP**	**AD**	**UI**
AA	**0.798**						
IA	0.689	**0.796**					
COL	0.637	0.643	**0.804**				
COM	0.597	0.572	0.637	**0.823**			
AP	0.691	0.684	0.617	0.649	**0.829**		
AD	0.561	0.608	0.644	0.558	0.578	**0.801**	
UI	0.585	0.639	0.574	0.628	0.688	0.502	**0.810**

**Table 5:** Loadings and cross loadings.

	**AA**	**IA**	**COL**	**COM**	**AP**	**AD**	**UI**
AA1	**0.850**	0.575	0.551	0.496	0.653	0.484	0.482
AA2	**0.778**	0.520	0.494	0.446	0.506	0.436	0.437
AA3	**0.761**	0.555	0.474	0.491	0.468	0.419	0.487
IA1	0.536	**0.792**	0.493	0.446	0.525	0.461	0.474
IA2	0.543	**0.808**	0.523	0.463	0.570	0.489	0.471
IA3	0.565	**0.786**	0.517	0.455	0.537	0.501	0.584
COL1	0.474	0.460	**0.786**	0.488	0.455	0.511	0.407
COL2	0.494	0.534	**0.819**	0.507	0.490	0.538	0.484
COL3	0.570	0.556	**0.806**	0.542	0.544	0.503	0.493
COM1	0.472	0.474	0.552	**0.829**	0.515	0.512	0.497
COM2	0.519	0.460	0.502	**0.827**	0.522	0.447	0.546
COM3	0.485	0.478	0.515	**0.813**	0.573	0.407	0.509
AP1	0.630	0.576	0.505	0.490	**0.842**	0.472	0.495
AP2	0.625	0.596	0.552	0.536	**0.842**	0.512	0.531
AP3	0.463	0.530	0.478	0.587	**0.804**	0.453	0.685
AD1	0.486	0.525	0.561	0.505	0.520	**0.825**	0.420
AD2	0.497	0.529	0.567	0.500	0.488	**0.845**	0.425
AD3	0.348	0.392	0.399	0.307	0.364	**0.729**	0.355
UI1	0.466	0.578	0.478	0.384	0.450	0.426	**0.721**
UI2	0.476	0.507	0.489	0.553	0.624	0.422	**0.851**
UI3	0.486	0.489	0.436	0.571	0.583	0.380	**0.851**

### The structural model

**Figure 2:**
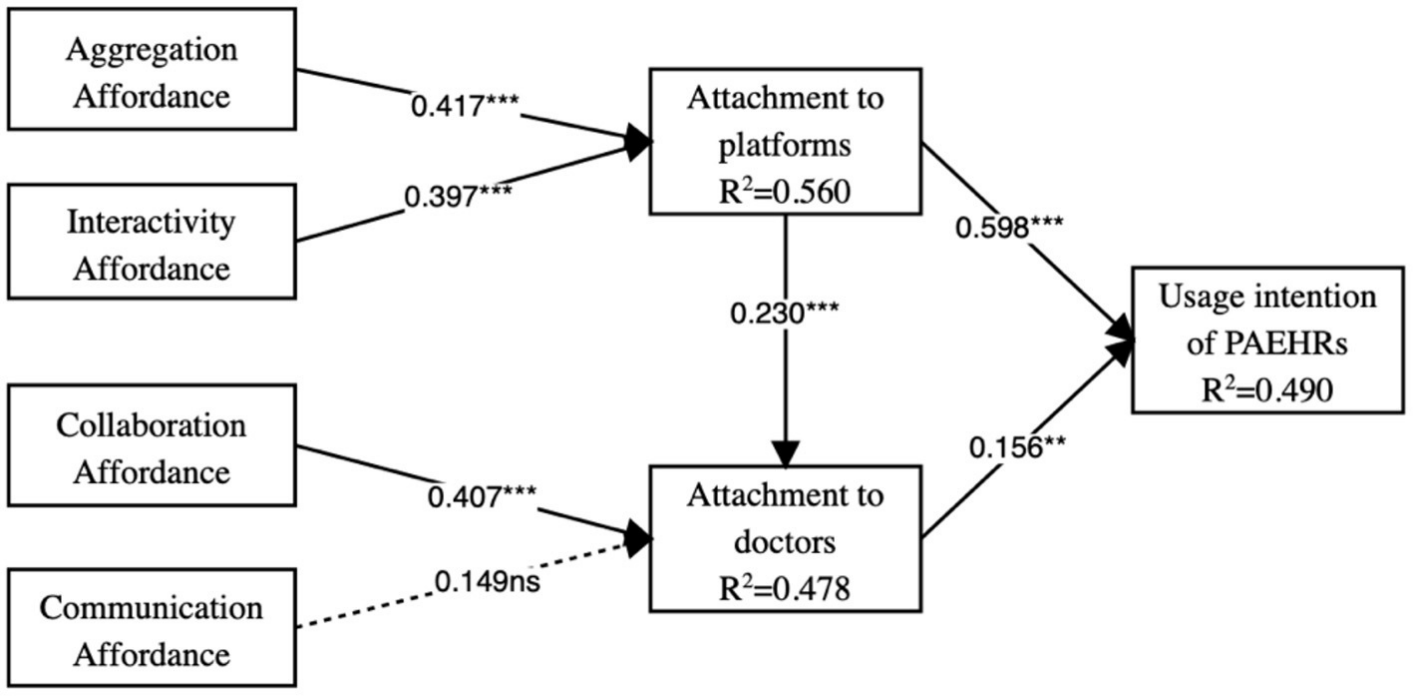
Structural equation model with standardized coefficients. **p < 0.01, ***p < 0.001, ns, nonsignificant. Nonsignificant paths are presented by a dashed line.

The test of the structural model included the examination of path coefficients and the corresponding significance levels. Figure 2 presents the results of the PLS analysis. The hypothesis testing results are listed in Table 6. We also adopted the bootstrapping technique that can directly test the influence of the independent variable on the dependent variable. The results show that the positive effects of attachment to PAEHRs' platform and doctors on older adults' usage intention are significant, supporting H1 and H2. Attachment to platforms significantly influences attachment to doctors, indicating that H3 is supported. Aggregation affordance and interactivity affordance significantly influence older adults' attachment to the PAEHR platform, and collaboration affordance significantly influences attachment to doctors, indicating that H4, H5, and H6 are supported. The results also suggest that the influences of communication affordance on attachment to doctors are not statistically significant. Hence, H7 is not supported.

**Table 6:** Results of hypothesis testing.

**Hypothesis**	**Paths**	**Path coefficients**	***t*-value**	***p*-value**	**Hypothesis validation**
H1	AP → UI	0.598	13.981	0	Supported
H2	AD → UI	0.156	3.145	0.002	Supported
H3	AP → AD	0.230	4.306	0	Supported
H4	AA → AP	0.417	8.804	0	Supported
H5	IA → AP	0.397	7.717	0	Supported
H6	COL → AD	0.407	6.063	0	Supported
H7	COM → AD	0.149	1.911	0.056	Not supported

We use a quantitative survey method to validate some hypotheses for the empirical study. In total, six of seven hypotheses were supported. In sum, these significant links indicate that PAEHRs' affordance and elders' usage intention of PARHEs have the relationships specified in the model.

## Discussion

This study investigated how older adults' attachment to platforms and doctors affects their usage intention of PAEHRs based on the view of affordance. For older adults, our findings show that their attachment to the platform and doctors of the PAEHR positively influences usage intention. Furthermore, older adults' objective attachment to the platform significantly influenced their emotional attachment to doctors on the PAEHR. Specifically, affordance about the aggregation and interactivity of PAEHRs is positively related to elders' attachment to the platform, and affordance about user-doctor collaboration is positively related to elders' attachment to doctors, while communication affordance has no significant effect on older adults' attachment to doctors.

### Facilitators from the perspective of attachment to platform

Drawing upon the affordance lens, we found that older adults' attachment to platforms and doctors can significantly improve their willingness to use PAEHRs. First, this study reveals that the aggregation affordance and interactivity affordance of PAEHRs are contributing factors for motivating older adults' attachment to the platforms. Specifically, with aggregation affordance, the PAEHRs' platform can integrate auxiliary functions in addition to electronic medical records, such as online consultation or health information seeking. The effect of integrated features can affect users' behaviors in the sociotechnical environment (48). Aggregation affordance provides older adults more options to use full-featured PAEHRs by combining different functions to achieve their health-related goals, which can contribute to their attachment to the platform. For example, the elders can enjoy a one-stop shop on PAEHRs, which is a blend of vicarious experience and direct communication with a professional physician for consultation. Depending on their condition, elder patients can choose whether to search for further health information, consult online, or go to an offline brick-and-mortar medical facility for further treatment based on the initial diagnosis provided by the online physicians.

Meanwhile, interactivity affordance allows users to interact with other actors or the environment where they operate through their actions (49). PAEHR's interactivity affordance affords older adults accessibility to their medical records and the permission to control personal preferences, update their health records or share their health-related opinions with their health care providers on the platform. ICT-enabled geriatric patient-friendly interactive experiences can largely enhance the attachment of older adults to PAEHRs, thus further promoting the positive use of health-related ICTs among older adults.

In addition, our results showed that older adults' attachment to the platform of PAEHRs has a positive relationship with their attachment to doctors and their usage intention of PAEHRs. Currently, people are increasingly inseparable from information technology, where it is difficult to distinguish which abilities are their own and which are endowed by ICTs. The functionally integrated PAEHR attracting users to communicate or collaborate with doctors on the platform is related to elders' attachment to the platform and causes their attachment to the doctor on the platform.

Moreover, the results also showed that attachment to the platform and attachment to doctors could both influence older adults' usage intention of PAEHRs, and attachment to the platform had a much more significant influence than attachment to doctors. This finding means that the PAEHR platform integrating functional and accessible features is the most critical factor that assures older adults have the ability to engage in activities on PAEHRs for health management.

### Facilitators from the perspective of attachment to doctors

Although PAEHRs' platform aggregates essential parts that attract older adults to use, some behavioral influence doctors might have is also critical for older adults' usage intention. We found that collaboration affordance positively influences older adults' attachment to doctors on the PAEHR, while communication affordance did not relate to this kind of attachment. Previous research has proven that communication is a success factor that can construct a harmonious doctor–patient relationship (50). As some studies have shown, doctors' communication and interpersonal skills are essential in health care (51). A good doctor–patient relationship contributes to patients' self-confidence and has a positive influence on therapeutic qualities (52). In the usage context of PAEHRs, users' attachment to doctors is dependent more on the ICT-based skills of patient-centered communication. We speculate that the reason this construct was not significant in this study may be related to the small percentage of older adults in our sample who had online consultations with their physicians (38%). Compared to other types of affordances, such as vicarious interactions, the number of elder patients who have direct communication with their physicians on PAEHRs is still a minority.

Furthermore, previous studies have reported that collaborative aspects were an essential factor influencing the patient–doctor relationship (37). This study also found that collaboration with doctors on the PAEHRs makes older adults feel attached to doctors. Consistent with this argument, prior work found that the patient's collaboration with health care providers promotes mHealth app use for the elders and empowers them with sustainable healthy lifestyle behaviors (7). According to the positive impact of collaboration affordance, cooperating with doctors on the PAEHRs platform helps elder individuals build dependency on the state of self-efficacy with the doctors' support. In particular, the treatments of geriatric diseases, especially chronic pain, are pressing problems for patients and their physicians, requiring older adults' long-term involvement in cooperation with doctors to solve their health problems. Through collaboration with doctors, the relationship between doctors and patients is also promoted, which generates users' attachment to doctors on the PAEHR platform.

This study has several theoretical implications. First, this study is unique in identifying the antecedents of older adults' attachment to doctors and platforms from the affordance lens. Second, this study established the connection between elders' involvement in health issues and attachment to the platform and doctors on PAEHRs, contributing to the literature on attachment theory in health-related ICTs. Our findings are useful for informing the development of optimal designs for elders-oriented applications to bridge the digital gap between older adults' endorsements and new forms of health-related ICTs.

Our research model of older adults' usage intention on PAEHRs has several practical implications. First, it offered healthcare organizations ideas on attracting older adults to engage in health management and improve health skills. Our research also called on doctors participating in online diagnosis and treatment to pay attention to their communication skills with patients. Second, we reminded health-related system designers to develop an accessible PAEHRs platform that aggregates various functions to support older adults in seeking health information, reading health-related articles, and sharing health opinions. Third, elder individuals' attachment to the platform can contribute to their attachment to doctors whom they can consult on health issues or collaborate on a medical plan. This result reminded PAEHRs' system design professionals that building an elders-oriented platform is the foundation on which supporting the collaboration between doctors and patients is worth being comprehensively considered.

## Conclusion

Elders' use of PAEHRs is an effective means of engaging and managing their health issues while also promoting the solution of aging problems. Our results revealed the inherent influencing mechanism of the affordance of PAEHRs on their attachment to doctors and platforms. In summary, designing the PAEHR more suitable for elder individuals, professionals need to focus leave space for patients' involvement in medical decision-making with their health care providers (14), which may also increase doctors' communication skills and patient compliance. Furthermore, PAEHRs should not only be equipped with electronic medical records and other assistive health-related functions to support collaboration between users and doctors but also guide doctors on the platform to pay attention to communication skills when cooperating with users, especially focusing on patient-centered communication, which can contribute to elders' attachment to doctors, thus promoting the usage intention of PAEHRs.

This research has limitations. First, we only focused on older adults who have experience using health-related apps, without considering elder individuals who cannot use mobile phones. Second, questionnaires were obtained online through the self-report of elders, which may lead to their recall bias. Third, the research questionnaire only investigated elder individuals in the form of text, which may not be conducive to their understanding. Although PAEHR applications have been globally implemented, there are many other health-related ICTs for elder individuals. Future research will include a wider range of users among the senior population, and researchers can study the elders-oriented design of other types of health-oriented products.

## Data availability statement

The raw data supporting the conclusions of this article will be made available by the authors, without undue reservation.

## Author contributions

XW conceived and designed the study, collected the data, did the data analysis, and wrote the first draft. YZ revised and edited the manuscript and supervised the manuscript. All authors contributed to the article and approved the submitted version.
